# Testing plant growth promoting microorganisms in the field - a proposal for standards

**DOI:** 10.3389/fpls.2023.1324665

**Published:** 2024-01-16

**Authors:** Daniel Neuhoff, Günter Neumann, Markus Weinmann

**Affiliations:** ^1^ Department Agroecology & Organic Farming, Institute of Crop Science and Resource Conservation, Rheinische Friedrich-Wilhelms-Universität Bonn, Bonn, Germany; ^2^ Department of Nutritional Crop Physiology (340h), Institute of Crop Science, University of Hohenheim, Stuttgart, Germany

**Keywords:** sustainable agriculture, crop yield and quality, experimental design, biostimulants, mode of action

## Abstract

In the European Union and worldwide there are a burgeoning markets for plant growth promoting microorganisms (PGPM) and other biological agents as soil improvers, bio-fertilizers, plant bio-stimulants, and biological control agents or bio-pesticides. Microbial agents have a major share in this development. The use of such products is often advertised with the promise of contributing to sustainable agricultural practices by increasing crop growth and yield and offering an alternative or substitute to decrease the dependency of agriculture on hazardeous agrochemicals. In contrast to registered microbial plant protection products, PGPM that are marketed in the EU as soil improvers or plant biostimulants, are not strictly required to have proven minimum efficacy levels under field conditions. Manufacturers only have to ensure that these products do not pose unacceptable risks to human, animal or plant health, safety or the environment. Uniform guidelines comparable to the EPPO - standards (European and Mediterranean Plant Protection Organisation) to test the efficacy in field trials are not available. This paper attempts to fill the gap. It proposes guidelines for PGPM field trial design and implementation, as well as recommendations for the type and scope of data collection and evaluation. Selected research papers from literature were evaluated to analyze, whether and to what extent the requirements are already met. The majority of the papers had a clear experimental design followed by proper data evaluation. Frequent deficiencies were the low number of tested environments and crop species, insufficient site and agronomic management description and missing data on soil humidity and temperature. Using the suggested standards is assumed to increase the expressive power of tested microbial products.

## 1 Introduction

Plant protection products (PPP) are subject to a demanding approval process including a testing of efficacy using EPPO standards (European and Mediterranean Plant Protection Organisation, Paris, France). Guidelines are available for a large range of specific indications, e.g. PP1/46(3) on efficacy evaluation of insecticides against wireworms (EPPO, 2023), or PP 1/002 (5) of fungicides against *Phytophthora infestans* on potato, foliar diseases on maize (PP 1/272 (1)), fungicides against *Gaeumannomyces graminis* causing take-all in cereal (PP 1/262 (1)), or criteria, as well as the experimental procedures, for determining the minimum effective dose of a plant protection product (PP 1/225(2)) (EPPO Bulletin, 2021b; EPPO, 2023).

Likewise, standards also exist for microbial plant protection products (PP1/276(1) published in EPPO Bullettin (2012), but without specific indications. Nowadays, various insecticides of microbial origin are well established on the market including entomopathogenic fungi such as *Beauveria bassiana* against locusts (Ranjan et al., 2021) or *Metarhizium brunneum* against wireworms (Brandl et al., 2017).

In addition to the market of strictly regulated microbial PPP, there is a burgeoning market for plant growth- promoting microorganisms (PGPM’s) and other plant biostimulants based on bioactive natural compounds. They are applied to plants with the aim to enhance nutrition efficiency, abiotic stress tolerance and/or crop quality traits without direct effects as fertilizers (Du Jardin, 2015; Weinmann and Neumann, 2020). As summarized by O’Callaghan et al. (2022) the use of these products is often advertised with the promise of contributing to sustainable agricultural practices by increasing crop growth and yield or reducing the demand for fertilizers and agrochemicals (e.g. Lantmännen BioAgri (2021), Agriges (2023); Corteva Agriscience (2023), Syngenta Biologicals (2023).

In Europe, the Regulation (EU) 2019/1009 (EU, 2023a), laying down rules on placing fertilizing products on the EU market, defines “plant biostimulants” as product with the function to stimulate plant nutrition processes independently of its nutrient content with the sole aim of improving one or more of the following characteristics of the plant or the plant rhizosphere: (i) nutrient use efficiency, (ii) tolerance to abiotic stress, (iii) quality traits, or (iv) availability of confined nutrients in the soil or rhizosphere.

The range of potential beneficial effects of living rhizosphere microorganisms, which implement their direct or indirect influence on plant performance by biological modes of actions, in particular those interfering with soil-plant-microbe interactions, is, however, scientifically well known to be much more multifaceted (Avis et al., 2008; Borriss, 2015; Weinmann, 2019).

Biostimulants have no effect against biotic stresses (e.g. pathogens and pests) and hence do not fall under the regulatory framework of pesticides. The list of biostimulants also includes PGPM such as N_2_-fixing bacteria genera (e.g. *Azotobacter*, *Azospirillum*, *Rhizobium*) or mycorrhiza fungi. Any PGPM marketed for crop production purposes must be registered as either PPP, biofertilizer or biostimulant and has to fulfil the corresponding specific requirements, as compiled for different categories of EU fertilizing products including microbial and non-microbial plant biostimulants in (**Table 1**).

**Table 1:** Categories of EU fertilizing products according to the REGULATION (EU) 2019/1009 and plant protection products according to the REGULATION (EC) No 1107/2009 in which plant growth promoting microorganisms (PGPM) and other biological agents for agriculture can be made available on the market.

Product Function Category (PFC)	Functional definition	Component material categories (CMCs)	Regulatory standards and product requirements
**EU Fertilising Products in general**	Providing plants or mushrooms with nutrient or improving their nutrition efficiency	Shall consist solely of component materials complying with the requirements for one or more of the CMCs listed in Annex II of REGULATION (EU) 2019/1009 including **CMC 7: Micro-organisms**	‘EU fertilising product’ means a fertilising product which is CE (European conformity) marked when made available on the market
**1. Fertiliser**	To provide nutrients to plants or mushrooms	**1. A: Organic Fertiliser:** shall contain organic carbon and nutrients of solely biological origin **1. B: Organo-Mineral Fertiliser:** co-formulation of 1. A and 1. C. **1. C: Inorganic Fertiliser:** shall contain macro- and/or micronutrients in inorganic form	Limits for contaminants (e.g. cadmium) and pathogens (e.g. *Salmonella*) Minimum contents for declared nutrients
**2. Liming Material**	To correct soil acidity	shall contain oxides, hydroxides, carbonates or silicates of the nutrients calcium (Ca) or magnesium (Mg).	Limits for contaminants (e.g. cadmium) Minimum neutralizing value, reactivity and grain size
**3. Soil Improver**	To maintain, improve or protect the physical or chemical properties, the structure or the biological activity of the soil	**3. A: Organic Soil Improver:** shall consist of material 95% of which is of solely biological origin (including peat, leonardite and lignite, but no other material which is fossilized or embedded in geological formations) **3. B: Inorganic Soil Improver:** other than an organic soil improver	Limits for contaminants (e.g. cadmium) and pathogens (e.g. *Salmonella*) Minimum contents for dry matter (20%) and organic carbon (7.5%) for organic soil improvers
**4. Growing Medium**	Products other than soil in situ, the function of which is for plants or mushrooms to grow in.	No further specification.	Limits for contaminants (e.g. cadmium) and pathogens (e.g. *Salmonella*)
**5. Inhibitors**	To improve the nutrient release patterns of a product providing plants with nutrients by delaying or stopping the activity of specific groups of micro-organisms or enzymes	**5. A: Nitrification Inhibitor** **5. B: Denitrification Inhibitor** **5. C: Urease Inhibitor**	20% reduction in the rate of ammoniacal nitrogen (NH_3_-N) oxidation, release of nitrous oxide (N_2_O), respectively hydrolysis of urea (CH_4_N_2_O), based on an analysis carried out 14 days after application at the 95% confidence level
**6. Plant Biostimulant**	To stimulate plant nutrition processes independently of the product’s nutrient content with the sole aim of improving one or more of the following characteristics of the plant or the plant rhizosphere: (a) nutrient use efficiency, (b) tolerance to abiotic stress, (c) quality traits, or (d) availability of confined nutrients in the soil or rhizosphere.	**6. A: Microbial Plant Biostimulant:** shall consist of a micro-organism or a consortium of micro-organisms, including dead or empty-cell micro- organisms and non-harmful residual elements of the media on which they were produced, which have undergone no other processing than drying or freeze-drying; and are referred to in CMC 7 in Part II of Annex II (i.e.: *Azotobacter* spp., Mycorrhizal fungi *Rhizobium* spp., *Azospirillum* spp.) **6. B: Non-Microbial Plant Biostimulant:** other than a microbial plant biostimulant	Limits for contaminants (e.g. cadmium) and pathogens (e.g. Salmonella) Shall have the effects that are claimed on the label for the plants specified thereon Shall have a pH optimal for contained micro-organisms and for plants.
**7. Fertilising Product Blend**	Product composed of two or more EU fertilising products of PFC 1 to PFC 6	Blending shall not change the nature of each component EU fertilising product and shall not have an adverse effect on human, animal or plant health, on safety, or on the environment …	Requirements of each component EU fertilising product in the blend has been demonstrated in accordance with the conformity assessment procedure applicable to that component EU fertilising product …

Similar to registered microbial and other PPP according to Regulation (EC) No 1107/2009 (EU, 2023b), also PGPM marketed as EU fertilizing products should be sufficiently effective and not pose a risk to human, animal or plant health, to safety or to the environment. While the obligatory and visible indicator that a EU fertilizing product including microbial plant biostimulant fulfills the safety requirements is the CE (European conformity) marking, the REGULATION (EU) 2019/1009 does not further specify the requirements for a sufficient efficacy assessment. General principles governing the CE marking and its relationship to other markings are set out in Regulation (EC) No 765/2008. Furthermore, information regarding the intended application method(s), effects claimed for each target plant, and relevant instructions related to the efficacy of the product should be given. This includes soil management practices, chemical fertilisation, incompatibility with plant protection products, recommended spraying nozzles size, sprayer pressure and other anti-drift measures, if applicable. For microbial plant biostimulant products in addition, all intentionally added micro-organisms shall be indicated (REGULATION (EU) 2019/1009).

However, elaborated guidelines for efficacy testing of PGPM used as plant biostimulants are so far not available in a comprehensive collection of standards for agronomic field experiments. Some general principles have already been suggested, but they rather focus on methods how to justify the claims of biostimulants for later submission to the admission authorities (Ricci et al., 2019). To prove such a claim the principles also allow the exclusive testing under controlled conditions. Other than the proposal here, they are not targeted on testing the practical agronomic benefit for farmers, although they include various important aspects, also considered here.

**Figure 1:**
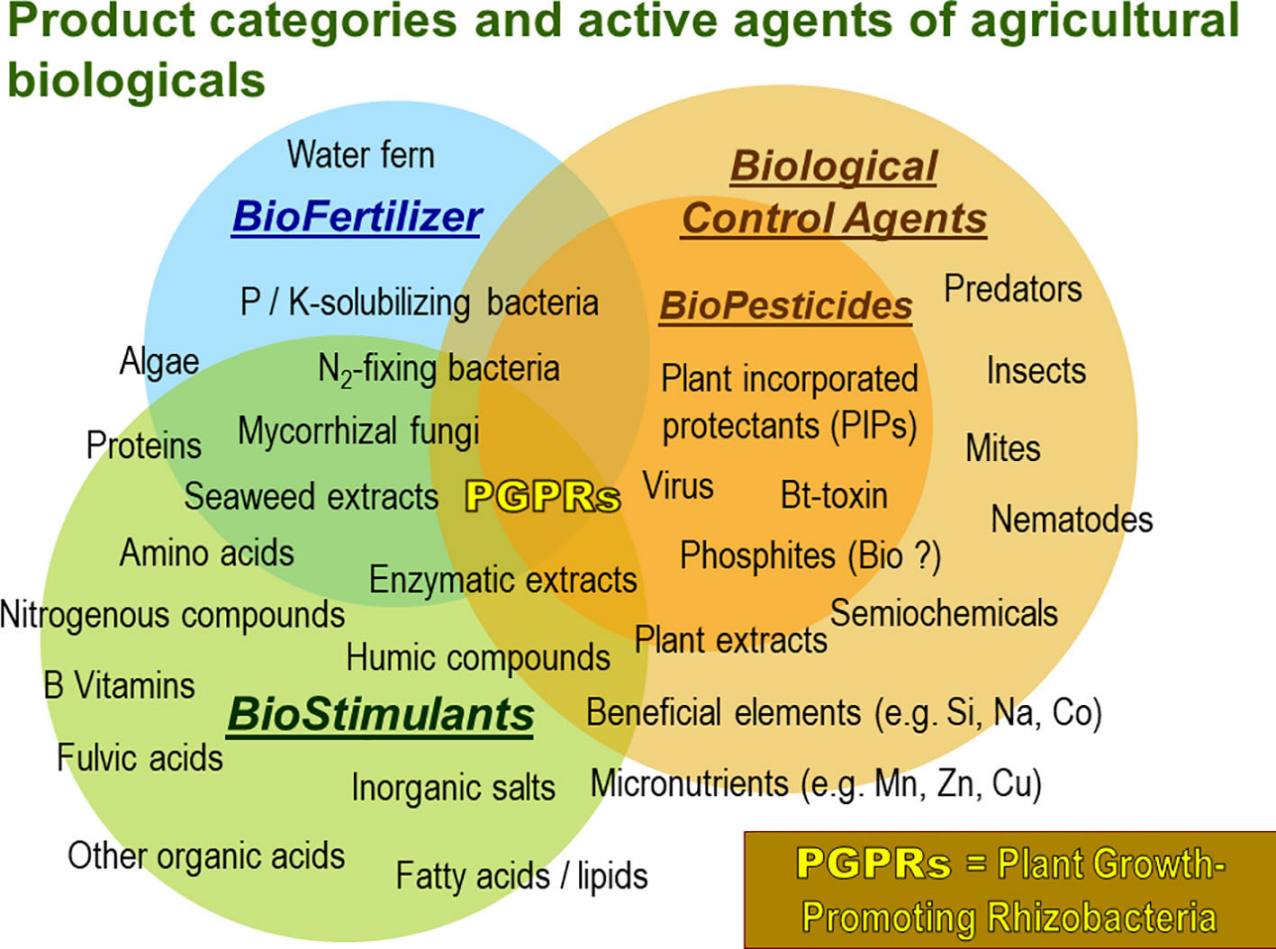
Product categories and respective types of active agents of agricultural biologicals for crop production. Especially microbial agents like plant growth promoting microorganisms (PGPMs) are characterized by multifaceted modes of actions. (Adapted from: Agricen Sciences’ analysis of market analysts, survey papers on Biostimulants, www.bpia.org).

At the same time the market for PGPM is continually growing offering a wide range of products of variable performance and often unspecified composition (**Figure 1**).

The best-known example for the successful use of PGPM in crop production are rhizobia bacteria, which live in endophytic symbiosis with leguminous plants (Herridge et al., 2008; Lindström and Mousavi, 2019) and have been first patented as plant inoculants already in 1896 (Nobbe and Hiltner, 1896). Plant growth-promoting rhizobacteria (PGPR) may also live in less specific associations with plant roots, potentially resulting in growth-promoting effects on crops. In tropical and subtropical soils, for example, species of the genus *Azospirillum* have been shown to effectively replace N fertilizer inputs by 25-50% (Fukami et al., 2018, Santos et al., 2019). In these cases, the mode of action has been mainly linked to an improved nitrogen supply to the legume crop resulting from rhizobial atmospheric nitrogen (N_2_) fixation. However, a wide range of other physiological mechanisms may affect crop growth as well. According to Hett et al. (2022), the potential functions of PGPM include (i) the facilitated acquisition of water and nutrients (primarily N, P, and Fe); (ii) the modulation of phytohormonal balances by changing the levels of auxins, cytokinins, gibberellins, abscisic, jasmonic and salicylic acids, mediating, inter alia, stimulation of root growth and modifications of plant development; (iii) the release of volatile organic compounds and siderophores with functions in stress priming and nutrient mobilization and (iv) the reinforcement of resistance against abiotic stress factors (Vessey, 2003; Martínez-Viveros et al., 2010; Glick, 2012; Glick, 2014; Vejan et al., 2016 and **Table 2**).

**Table 2:** Multifaceted effects of selected types of PGPMs as reported in the literature.

Type of PGPMs	Bio-stimulation, Bio-fertilization, Soil Improvement	Bio-Control, Plant Protection
*Pseudomonas* spp.	• Phytohormonal plant growth stimulation, N_2_-fixation and improved nutrient acquisition (Singh et al., 2023) • Solubilization of P and other sparingly soluble nutrients (Barin et al., 2022) • Promotion of mycorrhization (Viollet et al., 2017) and legume nodulation (Soussou et al., 2017) • Soil aggregation (Sandhya and Ali 2015) and metal detoxification (Balíková et al., 2022) by release of exopolysaccharides	• Competition for space and nutrients (Miftakhov et al., 2023) • Inhibition of pathogen growth by production of iron-binding siderophores like pseudobactin and pyoverdine (Srivastava et al., 2022) • Synthesis of antibiotic and antifungal compounds, such as 2,4-diacetylphloroglucinol (2,4-DAPG), (Zhang et al., 2020) • Induced systemic resistance in plants (Reshma et al., 2018)
*Bacillus* spp.	. Phytormonal growth stimulation, N_2_-fixation (Azeem et al., 2022) and triggering of stress responses in plants (Poveda and González-Andrés 2021) . Solubilization of P and other sparingly soluble nutrients (Saeid et al., 2018) . Promotion of mycorrhization, nutrient acquisition (Nanjundappa et al., 2019) and legume nodulation (Sibponkrung et al., 2020) . Soil aggregation (Deka et al., 2019) and heavy metal detoxification (Nazli et al., 2020) by release of exopolysaccharides	• Competition with pathogens for ecological niches and nutrients (Luo et al., 2022) • Production of secondary metabolites with antiviral, antibacterial, antifungal and nematicidal activity such as lipopeptide surfactins (Chowdhury et al., 2015; Chen et al., 2018) • Production of hydrolytic enzymes (e.g. chitinase, cellulose) with antagonistic activity against phytopathogens (Diabankana et al., 2022) • Induced systemic resistance in plants (Borriss et al., 2019; Miljaković et al., 2020)
*Rhizobium* spp.	• Phytormonal plant growth stimulation (Ferreira et al., 2020) • Symbiotic N2-fixation (Lindström and Mousavi, 2019) • Solubilization of sparingly soluble mineral nutrients, such as P and Zn (Verma et al., 2020) • Improved mycorrhization and increased number of newly formed mycorrhizal spores (Igiehon and Babalola 2021; Nasslahsen et al., 2022) • Production of phytohormonal compounds (indole acetic acid), exopolysaccharides and siderophores (Verma et al., 2020).	• Competition for nutrients, such for Fe as through siderophore production (Fahde et al., 2023) • Production of antibiotics, hydrocyanic acid (HCN), and hydrolytic enzymes (e.g. chitinase; Tamiru and Muleta, 2018) • Induced systemic resistance and enhance expression of plant defense-related genes (Díaz-Valle et al., 2019) • Multi-trophic plant-mediated antagonistic interactions among herbivores (aphids), pathogens (plant virus) and soil rhizobia (Basu et al., 2021)
*Trichoderma* spp.	• enhanced nutrient efficiency (Zin and Badaluddin, 2020)	• release volatile organic compounds (Joo & Hussein, 2022) • induced systemic resistance (Tahir et al., 2017) • competition with pathogens for ecological niches (El-Maraghy et al., 2020) • release of antifungal and antibacterial compounds (Khan et al., 2020
Arbuscular mycorrhizal fungi	• increased nutrient uptake (Nadeem et al., 2017 • buffering salinity effects (Saxena et al., 2017) • increased rooth growth (Wu & Zou, 2017) • higher drought resistance (Latef et al., 2016)	• induce the synthesis of plant signal substances (Schmitz & Harrison, 2014) • promote the synthesis of plant defense hormones (Schmitz & Harrison, 2014) • slowing down the process of roots infection by pathogens by morphological changes (Basyal & Emery, 2021)

**Figure 2:**
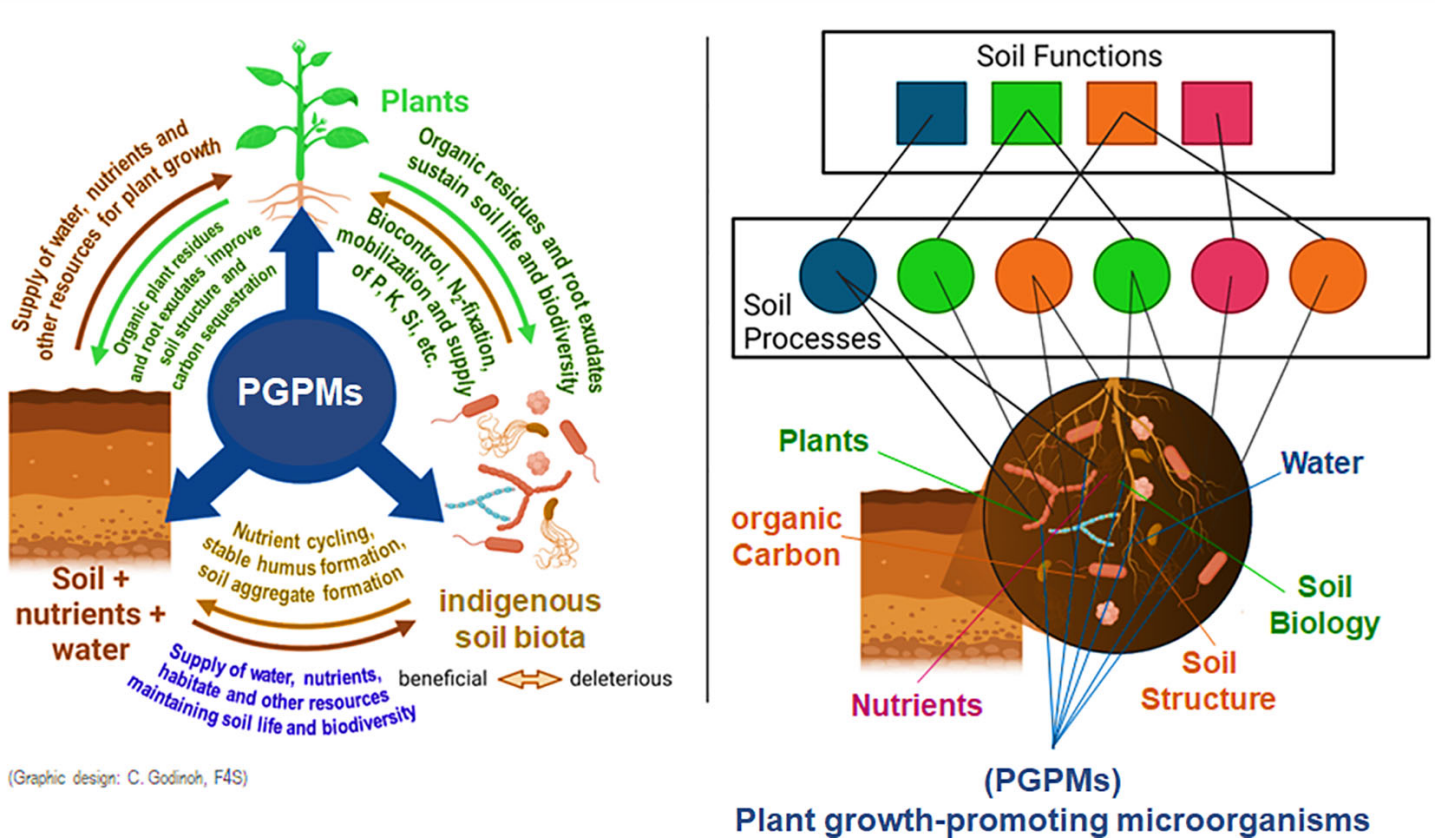
A better understanding of the synergies between soil, plants and soil life (Khati et al., 2020) and their impact on soil processes and ecosystem functions (Creamer et al., 2022) is of key importance for sustainable agriculture that is less dependent on the input of hazardeous agrochemicals by well-integrated PGPM applications (Weinmann and Neumann, 2020).

Furthermore, in non-axenic soil systems, introduced PGPM interact directly (e.g. antagonistic or synergistic modes of action) or indirectly via their influence on plant physiology (e.g, alterations in phytohormonal balances) or morphology e.g. by more intensive fine root and root hair formation (Avis et al., 2008; Calvo et al., 2017; Weinmann and Neumann, 2020). A schematic illustration of the numerous facets of PGPR interferences with soil-plant-microbial interactions is illustrated in **Figure 2**.

PGPM applications may affect various soil processes or plant physiology both expected to result in improved crop growth. However, the way from proven physiological effects of PGPM applications on plants to crop yield increases in the field is far.

Doubts on the general validity of plant growth promoting effects of microbial applications under field conditions have been raised repeatedly (Mayer et al., 2010, Meyer et al., 2019, Antoszewski et al., 2022; O’Callaghan et al., 2022). Following own experiments and published data, Hett et al. (2023) stated that there is often still no unequivocal evidence for the utility of PGPM in arable farming (Gouda et al., 2018; Santos et al., 2019; dos Santos Lopes et al., 2021).

Moreover, a so-called publication bias may reflect a certain disproportion between published results with positive effects and unpublished results with no effects. In addition, most studies have been carried out under controlled conditions in pot experiments. This type of trial is characterized by constant climatic conditions (temperature and soil humidity) and a limited soil volume. These factors significantly differ from field conditions and allow a more targeted control of environmental factors relevant for the expression of PGPM effects than field experiments. Interestingly, field experiments with missing microbial effects are rarely reported in the literature (Bashan et al., 2020), although there is increasing awareness that these results are of relevance for achieving a better understanding of the factors determining the field effectiveness of PGPM applications (Hett et al., 2023). Likewise, the number of positive reports from experiments under controlled environmental conditions is likely to provide an overly optimistic impression of the intrinsic potential effectiveness of tested agents (O’Callaghan et al., 2022). At the same time the importance of product formulations, integrated applications strategies and adapted soil and crop management strategies and other external influences for the expression of beneficial traits under practice conditions remains poorly understood (Römheld and Neumann, 2006).

Other authors, in contrast, take an optimistic view claiming that PGPM will increasingly help to make crop production more sustainable and see a great future of microbial biostimulants, despite the variable efficacy under field conditions (Santos et al., 2019; Sammauria et al., 2020; Shah et al., 2022; Singh et al., 2022). This is also reflected in the results of various forecasts on market share of biostimulants with a current global market size of approx. 3.3 Bn USD and predicted annual growth rates between 11 and 12% until 2030 (Fortune Business Insights, 2023).

In any case, a proper assessment of PGPM products under field conditions according to reproducible and comparable standards is an indispensable requirement prior to any recommendation for use in commercial farming. The validity of a study mainly depends on appropriate scientific standards including a robust experimental design (O’Callaghan et al., 2022). Here we outline a set of requirements that should be considered when testing PGPM efficacy in field trials.

## 2 Objectives and frame setting

Based on the challenges described above, the main objective of this paper is to propose uniform criteria for agronomic field trials suitable for scientifically testing the efficacy of PGPM based biostimulants offered for use in arable crops under temperate climate conditions. These trials are an important step prior to large-scale testing on fields with farmer equipment. Lab and pot experiments will not be considered here. These methodical tools with their controllable settings may significantly contribute to uncover distinct modes of action of a PGPM or to describe and quantify physiological processes induced by PGPM. They may also allow experimental screenings for preselection of promising microbial candidates for field testing, but they are far from being a proxy for field trials. Laboratory, pot and field experiments should carefully follow the standard rules of good experimental practice (EPPO Bulletin, 2021a). Key aspects to consider include the selection of a representative dosage suitable also for later field trials and the elimination of nutritional effects resulting from PGPM application by inclusion of appropriate controls.

A further objective of this paper is to sharpen the view of relevant stakeholders for possible sources of error, which may result in experimental artefacts and false conclusions. In contrast to EPPO standards for PPP the proposed standards are not binding for approval, but they may support producers and users to ensure a specific product quality for the benefit of all stakeholders.

In a first step we propose standards considered suitable for testing the efficacy of PGPM applications in field trials. In the subsequent discussion, we first justify the standards by underpinning them with evidence from literature. Finally, we compare the standards to the methodology described in published papers on PGPM field trials.

## 3 Testing the efficacy of PGPM

### 3.1 Field trials on efficacy

Trials need to be implemented by a skilled person with scientific aptitude. Experimenters should strictly adhere to the specifications given in the instructions with respect to crop specific mode and frequency of application, dosage and timing. They should not know treatment allocations in the field by doing a blind study. General principles of good experimental practice as outlined in EPPO guideline PP 1/181 [5] ,Conduct and reporting of efficacy evaluation trials including good experimental practice’ (EPPO Bulletin, 2021a) should be considered. These guidelines relate to various aspects including staff qualifications, use of suitable equipment and facilities, protocols, modes of operation and recording of results. In general, the following specific requirements for PGPM testing are less demanding than EPPO guidelines for PPP, since in contrast to them, results are not used for official registration purposes.

### 3.2 Crop and site selection

Selection of crop species as test plants should stick to the indications listed in the instructions. To rule out genotype specific treatment effects several cultivars may be tested. In general, a standard cultivar recommended for the region and already tested in published trials with other PGPM should be used. For specific indications, e.g. strengthening of plant vigor and stress tolerance, claim related choices including cultivars with known genotypic differences in stress resilience may be considered. The seed should be certified and not chemically treated unless otherwise specified. Crops should be grown on experimental plots with fairly homogenous distribution of soil properties and a known history for crop rotation and management to be performed as far as possible under *ceteris paribus* conditions. Field history refers to at least the last two preceding crops of the crop rotation including their management such as soil tillage, fertilization and crop protection. In situations where known gradients in soil properties and topographic factors cannot be avoided, adequate experimental designs like randomized block or Latin square can be used in order to statistically compensate for such limitation in site homogeneity. Experimental designs and statistical approaches for data evaluation that are intended to compensate for restrictions in randomization by mixed models containing both fixed and random effects need to be applied with care not to cover experimental artifacts. Optimally the persons performing the statistical data evaluations should be already involved in the planning and practical implementation of the field experiments to recognize early possible experimental biases that may lead to wrong statistical interpretations of the results.

Furthermore, a representative number of sites with well-described properties should be selected including soils with different levels of soil fertility, texture, pH, and levels of organic carbon (TOC). Selecting sites with different climatic conditions will also allow a quantitative assessment of effect stability. Annual replications may partly replace spatial variation with respect to variable weather (moisture, temperature). In case of limited test site availability, a priority might be set on testing products on less favorable soils, selected to avoid a masking of potential effects like improved root growth for better acquisition of sparingly available nutrient and water, which may not be discovered on very fertile and irrigated soils. If a specific function like improved phosphorus acquisition or enhanced tolerance to salinity stress is expected from the PGPM, site selection should consider this aspect: e.g. by conducting trials on low P or saline soils.

### 3.3 Treatment selection and mode of application

In the simplest case only one PGPM product is tested. In that case a minimum of two treatments would be needed (treated versus untreated). In addition to a negative control (= untreated), it is advisable to further include a positive control. Most suitable is another commercial product recommended for the same purpose. To avoid interference effects, in some cases, e.g. microbial products with additives, the inclusion of an autoclaved treatment or better the blank formulation without microbes should be taken into account. If specific effects, e.g. improved nitrogen supply or nutrient efficiency, are attributed to a PGPM - product, a second experimental factor, in that case mineral nitrogen application, should be included as a positive control, ideally in staggered treatments with lower and recommended rates. This approach allows the quantification of potential nitrogen effects resulting from PGPM application and hence the calculation of the nitrogen fertilizer equivalence (Delin et al., 2012).

Experimenters should strictly stick to the instructions on the application mode (e.g. seed coating) including dosage (in kg ha^-1^), timing and frequency of application. In some cases, however, it may be useful to include a treatment with higher dosage than prescribed to tickle out potential effects or to identify potential phytotoxicity. A quality check of PGPM products under use should be carried out shortly before trial implementation checking the viability of the inoculum.

### 3.4 Experimental design and trial implementation

The design of field trials with PGPM should be as simple as possible, i.e. a fully randomized or randomized complete block design. However, lateral contamination of adjacent plots must be avoided to obtain valid results. For that purpose, an untreated buffer stripe of at least 1 m between plots is needed. The adequate distance may depend on the type of test. For example, when spore forming pathogens (e.g. powdery mildews) or PGPM (e.g. Trichoderma fungi as mycoparasite of powdery mildews) are tested, adequate distances to obtain representative results may be found in respective EPPO guidelines.

Alternatively, this can be achieved by limiting the sampling area within a plot requiring larger plot sizes. A minimum of four field replications should be implemented, if possible, even six. Important variables to be assessed are crop yield and quality. Any other effect, except crop quality, is subordinated to yield and only becomes relevant if linked to a yield increasing effect or specific application target of the tested product (e.g. crop cultivation with less or without input of agrochemicals, while maintaining plant health, crop quality, yield level). For PGPM products which are labeled as phosphorus solubilizing bacteria for instance, empirical evidence has to be provided in field trials. Likewise, higher yields may either be additive to a given amount of fertilizer input, or substitutive, by reducing the input needed for a given yield level. Otherwise, PGPM effects such as early growth promotion or increased root growth may be advantageous, e.g. for crop competition against weeds or higher water and nutrient uptake. However, it remains to be shown, whether the observed effects sustain throughout the cropping season to produce a net benefit, or will later be compensated.

Root growth promotion may be a relevant criterion, even if aboveground biomass is not affected by PGPM application. However, complete excavation of root systems in fields, is highly demanding and labor intensive and associated with high risks of losing fine root structures. For a more standardized sampling it is possible to collect a representative number of cylinder-shaped soil cores of undisturbed soil at a defined distance and depth around the plants (Helmisaari and Brunner, 2006). Soil core samples collected under field conditions are frequently used for estimating rooting densities, specific root length and biomass or fine root distribution after root washing and digital image analysis as root growth and these variables may be influenced also by PGPM inoculants.

For proper assessment of yield data, a minimum of harvested plants and hence a sufficient plot size is needed (Stockem et al., 2022). According to EPPO Standards, representative plot sizes may vary between crop species and the tested effects (e.g. testing the effectiveness in plant protection against wind carried spore forming pathogens requires larger plot sizes than testing products for improved use efficiency of phosphorus fertilizers, since phosphorus has a low mobility in soils). Specific guidelines for the conduction of efficacy trials in wheat or other cereals are given in EPPO Standard PP1/026(4) and for maize in EPPO Standard PP1/272(2). According to these, net plot sizes for wheat and other cereals should be at least 10 m^2^. For maize, net plot size should be at least 2 rows x 10 m in length. The gross plot should have at least one additional treated border row on each side of the plot. With respect to accurate yield assessments it is recommended to take into account national standards for official testing of new varieties. In Germany, for instance recommended minimum plot size (harvesting area) for cereals including maize, oil seed rape and pulses such as faba bean is 10 m². Crops with lower planting density require higher minimum plots sizes, e.g. 12.5 m² for potatoes and 12 m² for sugar beet (Bundessortenamt, 2000).Whenever possible the plot size should be 15 m².

Trial establishment, in general by sowing or planting of the test plant and application of the treatments to be assessed, is of crucial importance and a relevant source of error. Good agricultural practices such as weather dependent activities, thorough seed bed preparation and optimal sowing machine settings must be considered and accurately recorded in the experimental protocol. Cross contamination of seed lots need to be avoided in any case, for instance when PGPMs are applied by seed treatment. Therefore, negative controls should be sown first. To avoid contamination of non-target plots, the sowing device should be cleaned or even be sterilized, e.g. by ethanol before applying next treatments. Seeds treated with pesticides may interfere with PGPM application and should only be used if explicitly mentioned in the instructions. In many cases it may be useful to cover the trials with bird protection nets to ensure uniform crop emergence.

### 3.5 Crop management

Experimental conditions and management practices can have a strong influence on the expression of beneficial PGPM traits and henceforth on the results of any trial to test their effectiveness. They need to be as close as possible to practical farming conditions. In some special cases, however, also treatments not covered by the standard practice need to be included. This applies i.e. for evaluation of stress-protective effects. For evaluation of drought stress experiments it might be necessary to use rain shelters or compare plant performance under rainfed vs. irrigated condition, [even in cases, where irrigation is not commonly used] using a drought sensitive variety. In general, the *ceteris paribus* approach must be followed. Some management options may interfere with PGPM application. When using mechanical weed control tools, for example, carry over effects of inoculum have to be avoided. If considered relevant precautionary measures need to be taken, such as disinfection of the working tools.

Crop management may interfere with potential mechanisms of PGPM either induced directly or indirectly. Most prominent is the direct use of pesticides that may harm the applied PGPM. Unless not specified in the product instructions at least the use of fungicides should be omitted in field trials. In a two factorial design pesticide application may be included as second factor, provided that the factor level ,no application’ is included. Likewise, tillage or fertilization intensity can affect the long-term performance particularly of fungal PGPM strains applied as soil inoculants.

The experimental protocol must include all information and dates of management practices from soil tillage, sowing, fertilization, weed control, pesticide application, irrigation and harvesting.

### 3.6 Data collection

It is essential to generate valid and consistent data, which help to explain the overall results. Therefore, assessments and measurements need to be carried out up from the beginning preferably from the same skilled persons. Blockwise assessments may help to avoid unsystematic errors, potentially resulting from a forced interruption of measurements, e.g. by rainfall. Treatments should not be known to the data collector during assessments. Whenever possible, published and approved methods should be used. Visual ratings, e.g. on plant vigor are important, but also a possible source of bias. An internal quality check of the rating quality might be considered. An easy way to do this is a repetition of the rating in random order followed by a statistical comparison of both data sets. Name of the data collector and date of collection need to be recorded in the experimental protocol. Any management measure, e.g. irrigation, and unusual events, e.g. abiotic stress, need to be recorded as well.

#### 3.6.1 Site (soil and climate)

Prior to the experiment soil texture should be assessed if unknown. Important soil chemical parameters include pH, soil organic matter and nutrient concentration in the topsoil (in general 0 – 30 cm). During the field trials soil temperature and moisture should be recorded in rooted soil horizon (e.g. 5 to 10 cm depth) at least during the two weeks following PGPM application. If no weather station is available on site, public data from a nearby weather station should be collected. In that case a rain gauge should additionally be installed at the trial.

#### 3.6.2 Crop growth

The phenological stages of the crop should be recorded. Non-destructive measurements such as plant height, stem diameter and NDVI (Pettorelli, 2013) are easy to collect and may give first hints of a growth promoting effect. For destructive measurements additional area is needed. Higher plant biomass (dry matter = dm) after oven drying at 60° C and shoot nutrient accumulation in kg ha^-1^ (= above ground biomass in kg dm ha^-1^ x % NPK in shoot dm) at a given phenological stage, often the beginning of flowering, can be an indicator for the effect of a PGPM. Depending on the crop density, i.e. plants per m², representative samples sizes may vary from 0.5 to 3 m². In general, the rule should be followed that the less plants per m² are sown (e.g. maize with 10 seeds per m² vs. wheat with 350 seeds per m²) the larger the sample area should be to compensate for genotypic effects not being related to the treatments.

The occurrence of pest and diseases needs to be regularly assessed in all plots. If available, EPPO guidelines, should be used, at least for the assessment of dominant pests and diseases.

#### 3.6.3 Crop yield and quality

Fresh and dry matter yield need to be collected from a representative (e.g. plot) area, in general a minimum of 15 m² is compulsory for a solid assessment. It is important to analyze the yield structure as well, since it may help to better explain the obtained results. In winter wheat trials, for example, the ear density, number of grains per ear and the thousand grain weight should be reported.

Price effective quality parameters should be assessed as well. For e.g. winter wheat basic quality parameters include crude protein and gluten content of the grains. The assessment of mycotoxin contamination such as DON can be considered in justified cases. Targeted quality assessments should only be carried out, when part of the product claims.

#### 3.6.4 Rhizosphere samples

Whenever possible rhizosphere samples should be taken in regular intervals. Metagenomic tools may help to quantify PGPM induced effects on the rhizobiome and ideally trace the fate of the inoculum after application including long-term effects on the soil microbiome and functional biodiversity. This, however, is a methodical challenge requiring considerable know-how and resources, which cannot be binding for standards.

### 3.7 Data evaluation and presentation of results

Prior to any statistical evaluation it is essential to check data for consistency and plausibility. The standard for field trial evaluation is Analysis of Variance (ANOVA) according to Fisher after testing the normal distribution of the residues and the homogeneity of the variances with standard tests. In general, a randomized complete block (RCB) design should be sufficient to compensate for potential field heterogeneity starting with a cross site evaluation. More sophisticated experimental designs should only be selected, if justified by an experimental factor. Testing the effect of PGPM applications on drought stress, for example, might require a split plot design with rain shelter plots. Given a sufficient number of data sets an effect size may be calculated (Hedges, 1982). Tests for mean comparisons depend on the research questions. Comparing several products among each other requires multiple comparison test such as the Tukey HSD-test. This robust *post-hoc* test can handle unequal sample sizes and variances, and controls the probability of making a type I error. If in specific cases individual treatments shall only be compared with an untreated control, the use of the Dunnett test may be considered.

Tables and figures need to be comprehensive allowing expert readers to quickly check statistical conditions. As a standard, both absolute and relative values for e.g. yield should be indicated. When showing efficacies against diseases, the absolute incidence level at least of the negative control should be indicated. Standard errors should be routinely reported as well.

## 4 Discussion

The guidelines for field trial testing of PGPM’s presented here are targeted on gaining valid results for arable crops in temperate climate. Following the guidelines can contribute to gaining realistic assessments of the practical relevance of a given PGPM product. They try to consider the cause effect relationships from both perspectives, i.e. the manufacturer and the user. A zero efficacy of a given PGPM application can be due to a fake product or to mistakes during production, storage or application. Avoiding the former protects the user, while the latter is relevant for both stakeholders.

### 4.1 Important factors ensuring the validity of field trial results

To exclude the use of products with insufficient performance it is important to have a quality check prior to application in field trials. Rapid screening tests under controlled conditions working with seedlings and young plants have been described in the literature (Akter et al., 2013) for pre-evaluation of the basic effectiveness of a given product. Moreover, according to the harmonized EU legislation (EU) 2019/1009, future registration of biostimulants will comprise CE certification and a documented experimental proof of efficiency.

A second important aspect is the fate of the inoculum after application. However, inoculant tracing under field conditions is not a task which can be easily integrated into routine field testing of PGPM products. It requires strain-specific DNA primers or PGPM strains carrying resistance factors against certain antibiotics which are not widely available for many products.

With respect to the experimental design key challenges are the selection of appropriate controls and the setting of minimum plot sizes for yield determination. Using an autoclaved control, or better a blank formulation may help to avoid side effects. Mayer et al. (2010) concluded on their four years experiments using also autoclaved EM (effective microorganisms) *that the small effects observed were not caused by the EM microorganisms but rather by the nutrient inputs derived from Bokashi.* However, even the use of autoclaved PGPM controls can induce plant responses independent of nutrient effects via modifications of rhizosphere microbial communities (Nassal et al., 2018). Likewise, autoclaving does not simply affect the active microbial agent from the product, but may alter other physical and chemical product features including the release of more or degraded cell components from the microbial agents that can still have bioactive effectiveness. Marmann et al. (2014) for instance reported that the antibiotic activity of living or autoclaved bacteria on other bacteria was similar.

Therefore, appropriate controls should rather consist of blank formulations without PGPMs either provided by the manufacturers or using the filtrates of liquid or suspended product formulations after removal of microbial cells via sterile filtration.

From an agronomic point of view, it is essential to ensure an accurate quantification of the crop yield and quality effect resulting from PGPM application. Yield may be the result of PGPM effects such as e.g. improved nitrogen supply, but improved nitrogen supply does not necessarily mean higher yield. The practical use of PGPM in arable crops is only justified if a proven benefit at least compensates for the product and application costs. This can be achieved with increases in crop yield or quality that allow for respective financial return or by improved use efficiency of fertilizers or other inputs that allow for respective cost savings. Relevance here means compensation of the product and application costs by additional revenues resulting from quality or yield increases or cost savings.

### 4.2 Further error sources

Soil factors can promote or restrict biological activity and effectiveness of PGPM inoculants (**Figure 2**). Soil pH and TOC (total soil organic carbon), but also available P and N pools have been identified as major drivers determining root traits and microbial community structures in soils (Lauber et al., 2009; Francioli et al., 2016). Accordingly, a recent meta-analysis reported that responsiveness to microbial phosphorous solubilizing PGPM and AMF inoculants decreases with increasing soil organic carbon content, whereas the response to microbial N_2_-fixers shows an opposite trend (Schütz et al., 2018). An increment of soil organic carbon status is reported to increase as well autochthonous populations of agronomically beneficial microorganisms, and may suppress deleterious or pathogenic microorganisms, which may be positively correlated with a higher microbial diversity (Francioli et al., 2016). This might in turn hamper the establishment or functional relevance of additionally introduced PGPM inoculants due to increased competition from the native microbial community (Paul, 2016) or decrease the need to improve soil health with additional PGPM products, because the soil indigenous PGPM already fulfil this task. In soils with high TOC content, also increased concentrations of humic substances may induce stimulating effects, which is well documented for this class of compounds (Jindo et al., 2020).

Soil pH can exert a direct selective effect on certain microbial taxa (Rousk et al., 2010) or indirectly affect plant-PGPM interactions via effects on nutrient availability in soils (Kemmitt et al., 2006; Neumann and Ludewig, 2023). All these factors need to be considered in both, experimental design and site selection, ensuring *ceteris paribus* conditions.

Using a field with an unknown history with respect to crop rotation, fertilization and crop protection may produce inconclusive results. For example, certain agronomic practices such as applications of fungicides or glyphosate-based herbicides, intensive tillage and fertilization or crop rotations with non-mycotrophic pre-crops, can significantly inhibit the establishment and growth of mycorrhizal associations and induce harmful alterations in the soil microbiome (e.g. predomincance of phytopathogens). These processes can also interfere adversely with arbuscular mycorrhizal fungi (AMF) applied as biostimulants (Oehl et al., 2004; Oehl et al., 2005; Helander et al., 2018; Sommermann et al., 2018). However, compatibility of PGPM products with various pesticides should be usually indicated by the application instructions provided by the manufacturers.

Missing effects of PGPM application may also be due to a masking effect resulting from high nutrient availability either in pots (Hett et al., 2022) or in the field (Hett et al. 2023) and limited impact of stress factors. On the other hand, also extreme nutrient limitations or stress factors affecting root growth and activity particularly during the sensitive establishment phase of PGPM inoculants in the rhizosphere can counteract or limit the expression of beneficial effects on the host plants. Accordingly, on soils with limited nutrient availability a starter fertilization with P and N can exert a beneficial impact on the establishment of arbuscular mycorrhizal associations or the symbiosis with N_2_-fixing bacteria (Bittman et al., 2006; Chekanai et al., 2018). In line with these findings also a recent meta-analysis revealed the highest efficiency of bacterial inoculants supporting plant P acquisition on soils with moderate P availability, while the benefits declined at higher and lower P levels (Schütz et al., 2018). Moreover, recent literature surveys suggest that P-solubilizing microorganisms used as inoculants contribute to the P nutrition of the host plant primarily through their long-term impact on nutrient cycling via release of sequestered P from decaying microbial biomass, rather than providing P by direct nutrient solubilization in the rhizosphere (Raymond et al., 2021).

Apart from the fertilizer dosage, also the form of fertilizer supply can affect PGPM performance. Numerous studies have reported positive PGPM effects in combination with N-rich fertilizers based on animal waste products (manures, guano, meat-, hair and feather meals) inoculants such as *Bacillus*, *Pseudomonas*, and *Trichoderma* (Thonar et al., 2017; Mpanga et al., 2018; Bradáčová et al., 2020; Behr et al., 2023). Particularly on soils with low TOC content, the use of these fertilisers might improve the carbon supply to the fast-growing copiotrophic inoculants, alongside with a starter fertilization effect promoting rhizosphere establishment. Also, the form of nitrogen supply (nitrate vs. ammonium) can affect plant-PGPM interactions. Particularly on soils with limited P-availability, ammonium-dominated fertilization promoted the acquisition of sparingly-soluble P sources and other nutrients in combination with various fungal and bacterial PGPM inoculants based on strains of *Bacillus*, *Paenibacillus*, *Pseudomonas*, *Trichoderma* and *Penicillium* (Mpanga et al., 2018; Mpanga et al., 2019a; Mpanga et al., 2019b).

PGPM products should be screened for potential non-microbial compounds that may have plant growth promoting effects as well, e.g. micronutrients or other biostimulants such as seaweed extracts or humic acids frequently applied together with PGPM in combination products. However, even pure microbial products can contain formulation additives possessing a certain biostimulatory potential. This applies e.g. for protein-based additives, such as milk powder or soybean protein which may liberate bioactive peptides (Colla et al., 2015) during degradation in the rhizosphere.

### 4.3 Comparison of the proposed standards with methods in published research

In total, 18 research papers were selected and checked for their conformity to the suggested standards. Paper selection criteria included the testing of an arable crop in field trials preferably under temperate climatic conditions excluding experiments with Rhizobia and on salt stress. Some 22 methodical criteria were checked. The majority of the papers fulfilled important parts of the criteria including experimental design, PGPM application technique and statistical evaluation (**Table 3**). However, important methodological details such as information on field history and crop management, but also data on soil humidity and temperature were rarely reported. Half of the studies did not check the quality of the inoculum prior to sowing. In most cases, the number of tested environments was low (n =2) and only one crop was tested. At least some recent studies (n = 5) traced the fate of the inoculum after application (Tab 3).

**Table 3:** Consistency of PGPM field trial methodology with a range of criteria based on ten published research papers.

running number *	1*	2	3	4	5	6	7	8	9	10	11	12	13	14	15	16	17	18
publication year 20–**	22	20	21	22	23	23	19	10	23	21	16	20	15	20	06	15	01	20
type of product	d	d	d	d	d	d	b	c	c	d	c	d	d	c	d	b	d	c
Number of products tested (n)	3	1	3	5	3	1	4	3	2	1	2	2	1	4	7	1	1	1
test crops (n)	1	1	1	1	1	1	1	4	1	1	1	1	1	1	1	1	1	1
number of tested environments (n)	2	2	2	1	3	2	1	1	2	1	2	2	2	2	2	92	2	2
indications on field history (**Y**es/No)	N	N	N	N	**Y**	**Y**	N	**Y**	N	N	**Y**	N	N	N	N	N	**Y**	N
negative control (yes/no)	**Y**	N	**Y**	**Y**	**Y**	**Y**	**Y**	**Y**	**Y**	**Y**	**Y**	**Y**	**Y**	**Y**	**Y**	**Y**	**Y**	**Y**
positive control (yes/no)	N	**Y**	N	N	**Y**	N	**Y**	N	**Y**	N	**Y**	N	**Y**	**Y**	**Y**	**Y**	**Y**	N
autoclaved or blank control (yes/no)	N	N	N	N	N	N	N	**Y**	N	**Y**	N	N	N	N	N	N	N	N
experimental design indicated (**Y**es/No)	**Y**	**Y**	**Y**	**Y**	**Y**	**Y**	**Y**	**Y**	**Y**	**Y**	**Y**	**Y**	**Y**	**Y**	**Y**	**Y**	**Y**	**Y**
number of field replications (n)	3	5	4	4	4	4	4	4	4	3	5	3	4	4	4	6	5	4
plot size (m²)	4,8	9,6	400	15	30	3	n.i.	n.i.	24	2.5	45	13	9.6	29	13	30	15	15
clear indication of dosage (**Y**es/No)	**Y**	N	**Y**	N	**Y**	**Y**	N	**Y**	**Y**	**Y**	**Y**	**Y**	**Y**	**Y**	**Y**	**Y**	**Y**	**Y**
booster dose	**Y**	N	N	N	**Y**	N	N	N	N	**Y**	**Y**	N	N	N	N	N	N	N
quality check of product (**Y**es/No)	**Y**	N	**Y**	N	**Y**	**Y**	N	**Y**	N	**Y**	**Y**	**Y**	N	N	**Y**	N	N	N
plot size for yield quantification (m²) ***	n.i.	9,6	3	n.i.	3	0,5	n.i.	n.i.	n.i.	2.5	24	n.i.	n.i.	5.4	n.i.	30	n.i.	15
field tracing of inoculant	N	N	**Y**	N	N	**Y**	**Y**	N	**Y**	N	N	N	**Y**	N	N	N	N	N
Number of para-meters assessed (n)	>5	>5	>5	<5	>5	>5	>5	>5	>5	>5	>5	>5	>5	>5	>5	<5	>5	<5
pesticide application (**Y**es/No/not indicated)	N	n.i.	N	n.i.	N	Y&N	**Y**	N	N	n.i.	n.i.	**Y**	n.i.	**Y**	n.i.	n.i.	**Y**	**Y**
chemical soil data (**Y**es/No)	**Y**	**Y**	**Y**	**Y**	**Y**	N	**Y**	**Y**	N	**Y**	**Y**	**Y**	**Y**	**Y**	**Y**	N	**Y**	N
physical soil data (**Y**es/No)	**Y**	N	N	N	**Y**	**Y**	**Y**	**Y**	**Y**	N	N	N	N	**Y**	N	N	N	N
soil humidity and temperature data	N	N	N	N	**Y**	N	N	N	N	N	Y	N	N	N	N	N	N	N
clear indication of statistical (**Y**es/No)	**Y**	**Y**	**Y**	**Y**	**Y**	**Y**	**Y**	**Y**	**Y**	**Y**	**Y**	**Y**	**Y**	**Y**	**Y**	**Y**	**Y**	**Y**

*: 1 = Gopalakrishnan et al., 2022; 2 = Ye et al., 2020; 3 = Nacoon et al., 2021; 4 = Frezarin et al., 2023; 5 = Hett et al., 2023; 6 = Behr et al., 2023; 7 = Bradáčová et al., 2019; 8 = Mayer et al., 2010; 9 = Symanczik et al., 2023; 10 = Mukherjee et al., 2021; 11: Nkebiwe et al., 2016; 12: Bakhshandeh et al., 2020; 13: Cai et al., 2015; 14: Gabre et al., 2020; 15: Çakmakçi et al., 2006; 16: Leggett et al., 2015; 17: Vessey and Heisinger, 2001; 18: Fröhlich et al., 2012.

**: commercial (c), development (d), b = both, ***: n.i. = not indicated means either plot size or smaller.

n.i. = not indicated.

Due to missing clarity of some of the criteria some arbitrary decisions may limit the specific validity of the evaluation. However, the rough evaluation suggests that a fulfilment of the listed criteria remains critical, even if the scientific quality of the selected papers tended to be high. Even though there are no prizes to be won for just publishing detailed data on crop yields and explaining management factors, they should be integrative part of future field research on PGPM.

## Author contributions

DN: Conceptualization, Writing – original draft. GN: Methodology, Writing – review & editing. MW: Visualization, Writing – review & editing.
